# EphA3-targeted chimeric antigen receptor T cells are effective in glioma and generate curative memory T cell responses

**DOI:** 10.1136/jitc-2024-009486

**Published:** 2024-08-06

**Authors:** Leesa Lertsumitkul, Melinda Iliopoulos, Stacie S Wang, Sarah J McArthur, Lisa M Ebert, Alexander J Davenport, Raelene Endersby, Jordan R Hansford, Katharine J Drummond, Ryan Cross, Misty R Jenkins

**Affiliations:** 1 Immunology Division, The Walter and Eliza Hall Institute of Medical Research, Melbourne, Victoria, Australia; 2 Department of Medical Biology, The University of Melbourne, Parkville, Victoria, Australia; 3 The Royal Children's Hospital Melbourne, Melbourne, Victoria, Australia; 4 Translational Oncology, Centre for Cancer Biology, Adelaide, South Australia, Australia; 5 The University of Adelaide Adelaide Medical School, Adelaide, South Australia, Australia; 6 Cancer Clinical Trials Unit, Royal Adelaide Hospital, Adelaide, South Australia, Australia; 7 Brain Tumour Research Program, Telethon Kids Institute, Perth, Western Australia, Australia; 8 Michael Rice Children’s Hematology and Oncology Center, Women’s and Children’s Hospital; South Australia Health and Medical Research Institute; South Australia ImmmunoGenomics Cancer Institute, University of Adelaide, Adelaide, South Australia, Australia; 9 Department of Neurosurgery, Royal Melbourne Hospital Department of Surgery, Parkville, Victoria, Australia; 10 Department of Surgery, The University of Melbourne, Parkville, Victoria, Australia; 11 Department of Biochemistry and Chemistry, La Trobe University, Melbourne, Victoria, Australia

**Keywords:** Immunotherapy, Chimeric antigen receptor - CAR

## Abstract

**Background:**

High-grade gliomas including glioblastoma (GBM) and diffuse midline gliomas (DMG) represent the most lethal and aggressive brain cancers where current treatment modalities offer limited efficacy. Chimeric antigen receptor (CAR) T cell therapies have emerged as a promising strategy, boasting tumor-specific targeting and the unique ability to penetrate the blood-brain barrier. However, the effective clinical application hinges on the optimal choice of antigen, with a limited number, currently under investigation.

**Methods:**

We employed cell surface proteomic analysis of primary human high-grade glioma samples from both adult and pediatric patients. This led to the identification of Ephrin type-A receptor 3 (EphA3) as a prevalently expressed target. We engineered a second-generation EphA3-targeted CAR T cell and assessed function using in vitro and in vivo models of GBM and DMG.

**Results:**

EphA3-targeted CAR T cells demonstrated robust antigen-specific killing of human GBM and DMG cell lines in vitro. In an orthotopic xenograft NSG mouse model, EphA3-targeted CAR T cells not only effectively eradicated tumors but also established a functional T cell population protective on rechallenge. Remarkably, mice rechallenged with a second contralateral orthotopic tumor implantation achieved complete tumor clearance and maintained a sustained complete response 6 months following initial treatment.

**Conclusion:**

Building on the proven safety profile of EphA3 antibodies in clinical settings, our study provides compelling preclinical evidence supporting the efficacy of EphA3-targeted CAR T cells against high-grade gliomas. These findings underscore the potential for transitioning this innovative therapy into clinical trials, aiming to revolutionize the treatment landscape for patients afflicted with these formidable brain cancers.

**WHAT IS ALREADY KNOWN ON THIS TOPIC**

- High-grade gliomas such as glioblastoma and diffuse midline glioma are incurable, with current therapies proving largely ineffective. Chimeric antigen receptor (CAR) T cell therapies have shown promise due to their specificity and ability to cross the blood-brain barrier, but the selection of effective antigens remains a critical hurdle, limiting their clinical advancement.

**WHAT THIS STUDY ADDS**

- This study identifies Ephrin type-A receptor 3 (EphA3) as a highly expressed antigen in both adult and pediatric high-grade gliomas and demonstrates the potent efficacy of EphA3-targeted CAR T cells in vitro and in vivo. The findings provide robust preclinical evidence supporting the potential of EphA3-targeted CAR T cells to significantly improve outcomes for high-grade glioma patients.

**HOW THIS STUDY MIGHT AFFECT RESEARCH, PRACTICE, OR POLICY**

- The demonstration of EphA3 as a viable target for CAR T cell therapy in high-grade gliomas can guide future research and clinical trial design, potentially shifting current treatment paradigms. This study advocates for the inclusion of EphA3-targeted CAR T cells in the therapeutic arsenal against high-grade gliomas, paving the way for regulatory considerations and adoption into clinical practice.

## Introduction

 High-grade gliomas are severe brain cancers with a very poor prognosis, affecting both adults and children, though distinctly different diseases. Glioblastoma (GBM) is the most prevalent and agressive in adults. Globally, GBM has an incidence rate of 0.59–5 per 100,000 with increasing trends noted across multiple countries.^1^ The median survival time postdiagnosis ranges from 6 to 15 months despite aggressive treatments including surgery, chemotherapy, and radiation. These tumors typically manifest in the cerebral hemispheres which manage essential neurological functions such as motor control, language, and cognition. This localization complicates achieving complete surgical removal and managing postoperative relapse, which typically occurs 6–9 months post primary treatment.^2 3^ In contrast, pediatric patients mainly suffer from diffuse midline glioma with Histone 3 K27 mutated (DMG, H3K27), a fatal and untreatable condition. These tumors develop in the brainstem and other central structures of the brain, regions where surgical interventions are not feasible. The treatments available, including radiation and chemotherapy, pose severe systemic toxicities particularly detrimental to the developing brains of children and adolescents. Typically, survival does not extend beyond a year from diagnosis.^4^ The urgent need for innovative treatments remains critical to potentially enhance both survival and quality of life for these young patients with high-grade glioma.

The complexity of treating brain tumors stems from their heterogeneity exemplified by the diverse cellular composition, genetic mutations, and variations in the microenvironment. While immune therapies targeting programmed cell death protein 1 (PD-1) and cytotoxic T lymphocyte associated protein 4 (CTLA-4) have improved outcomes in other cancers such as renal cell carcinoma (RCC), non-small cell lung cancer (NSCLC), and melanoma, these approaches have not yielded similar successes in brain cancers due to their unique resistance mechanisms.^5 6^ This highlights the necessity for a multi-targeted therapeutic approach that addresses multiple antigens simultaneously to reduce the potential for tumor resistance and relapse, thus paving the way for more effective management of this challenging disease.

Chimeric antigen receptor (CAR) T cell clinical trials targeting GBM antigens such as EGFRvIII,^7^ IL13Rα2,^8^ in adults, and also B7-H3 (CD276) for diffuse intrinsic pontine glioma (DIPG)^9^ and human epidermal growth factor receptor 2 (HER2) in central nervous system (CNS) tumors, including DMG,^10^ have confirmed the safety of these cellular therapies in treating brain cancer. However, with limited efficacy observed to date, the focus is shifting to integrating additional novel targets to combat tumor heterogeneity.

A recent interim phase I clinical report administering CAR T cells delivered by Ommaya reservoir directly into the brain have demonstrated transient antitumor responses in adult patients using armored CAR T cells targeting EGFRvIII, but secreting an EGFR-CD3 bispecific antibody directly into the tumor site in three cases of adult GBM.^11 12^ In addition, a recent report on the first DMG patients treated with GD2-targeted CAR T cells revealed clinical and radiographic improvement efficacy with no toxicity, underscoring the promise of the approach.^13^ The evaluation of new targets for CAR T cell therapies is therefore crucial to enable progress towards successful clinical outcomes.

Ephrin type-A receptor 3 (EphA3) is a member of the largest family of tyrosine kinase receptors. Eph receptors are involved in various cellular processes including embryonic development, tissue homeostasis, and disease progression. Mutations in EphA3 have been well characterized in multiple cancers including lung,^14^ colon,^15^ melanoma, and glioblastoma.^16^ EphA3 has also been identified as highly expressed in leukemia,^17^ melanoma,^18^ and bladder, colorectal, esophageal, gastric, and prostate cancers, and particularly high expression in liver cancer.^19^ Its role in tumor progression has been noted with expression in stromal cells and tumor microenvironment of various solid cancers.^20^ EphA3 was shown to be overexpressed in 40% of GBM patients’ samples, with high expression in glioma stem cells, allowing tumors to remain in an undifferentiated and tumorigenic state.^21^ Importantly, multiple studies have demonstrated a lack of EphA3 expression in normal healthy brain,^22^ and a lack of expression on vital organs including the heart, liver, kidney, and lung^23^ strongly suggesting that off-tumor on-target effects are unlikely.^17^

A humanized anti-EphA3 antibody (ifabotuzumab) has shown specific tumor targeting in early phase clinical studies for hematologic malignancies^24^ (NCT01211691) and GBM (NCT03374943) reinforcing its potential as a therapeutic target. No detectable uptake into normal tissues via whole-body distribution imaging was demonstrated with radiolabeled ifabotuzumab in 12 patients with recurrent GBM.^25^ Preclinical development of an antibody drug conjugate and radiolabeled antibody using an earlier iteration of ifabotuzumab (Clone IIIA4) has demonstrated antitumor responses compared with a naked antibody control in an orthotopic GBM model, but no long-term responses suggesting that improvements to the therapeutic development can be made.^26^

Here, we explore the preclinical efficacy of EphA3-targeted CAR T cell therapy in GBM and DMG. Employing a second-generation CAR construct, we demonstrate potent and antigen-specific in vitro cytotoxicity and in vivo tumor eradication in intracranial brain tumor models. This study pioneers a new therapeutic avenue with the prospect of significantly ameliorating outcomes in high-grade glioma patients, highlighting a ground-breaking leap towards clinical translation.

## Materials and methods

### Patient and public involvement statement

While the current study is preclinical in nature, focusing on the development of EphA3-targeted CAR T cell therapies for high-grade gliomas, our research program is deeply committed to involving patients and the public in our work. We understand the value of patient and consumer insights in guiding research that is not only scientifically sound but also aligned with the needs and experiences of those it aims to benefit. To this end, we have established a patient advisory panel consisting of individuals who have been affected by high-grade gliomas, either directly or as caregivers. This panel provides feedback on all aspects of our research program, including study design, potential impact, and the dissemination of our findings. Their involvement ensures that our work remains patient-centered and is communicated in a manner that is accessible and meaningful to the wider community.

### Cell lines and cell culture

The human U87 cell line was kindly provided by Rodney Luwor (Royal Melbourne Hospital, The University of Melbourne). The human H4, SW1088, U118, and A172 cell lines were obtained from the American Type Culture Collection (ATCC). The U251-mCherry-Luciferase (U251CL) cell lines were kindly provided by Francine Ke (WEHI). HEK293T cells were obtained from within WEHI. D283 human medulloblastoma cells were gifted by Darell Bigner^27^ (Duke University) and PER547 were generated at Telethon Kids Institute.^28^ STR analysis confirmed the identity of all cell lines. Cell lines were maintained at 37°C, 5% CO_2_ in RPMI-1640 medium (WEHI) supplemented with 10% fetal calf serum (FCS, Bovogen), 2 mM glutamine (Gibco), 100 U/mL penicillin, and 100 µg/mL streptomycin (Gibco), except for D283 that were maintained in Dulbecco’s Modified Eagle Medium (DMEM) supplemented as above.

Patient-derived K27M mutated DMG models using the previous nomenclature DIPG including, SU-DIPG4, −13 to –17, −19 to –21, −25 to –27, −33 to –35, −36, were a generous gift from Professor Michelle Monje (Stanford University School of Medicine).^29^ DMG cells were grown in media at a 1:1 ratio of DMEM/F-12 (Invitrogen) and Neurobasal-A Medium (Invitrogen) supplemented with 10 mM HEPES (Gibco), 1 mM sodium pyruvate (Gibco), 0.1 mM Minimal Essential Media (MEM) non-essential amino acids (NEAA, Gibco), 2 mM GlutaMAX-I (Gibco), antibiotic-antimycotic (containing 100 units/mL of penicillin, 100 ug/mL of streptomycin, 0.25 ug/mL of Gibco Amphotericin B (Gibco), B27 supplement (Invitrogen), 20 ng/mL epidermal growth factor (EGF, Shenandoah Biotech), 20 ng/mL fibroblast growth factor (FGF, Shenandoah Biotech), 10 ng/mL platelet-derived growth factor (PDGF)-AA, (Shenandoah Biotech), and 10 ng/mL PDGF-BB (Shenandoah Biotech), and 2 µg/mL Heparin (StemCell Technologies). Pediatric DMG cell line (SU-DIPG36) was transduced with a lentivirus encoding green fluorescent protein (GFP) firefly luciferase (pFUGW-Luc-IRES-GFP) as previously described.^30^ All cells were verified as mycoplasma negative by polymerase chain reaction (PCR) analysis at the WEHI internal facility and were cultured and maintained at 37°C in 5% CO_2_.

Primary human peripheral blood mononuclear cells (PBMCs) were obtained from the Australian Red Cross (Agreement #23-06VIC-16). Human T cells isolated from the PBMCs were maintained in RPMI (WEHI) enriched with 10% FCS, 1 mM sodium pyruvate (Gibco), 2 mM GlutaMAX-I (Gibco), 0.1 mM NEAA (Gibco), 50 µM Beta-mercaptoethanol (Sigma), 100 U/mL penicillin (Gibco), and 100 µg/mL streptomycin (Gibco), and 50 IU/mL rhIL-2 (Peprotech, #200–02).

### Primary human tissue

For EphA3 immunohistochemistry analysis, tumor tissues were obtained through SA Pathology as archival diagnostic specimens. Their use was approved by the Central Adelaide Local Health Network Human Research Ethics Committee (approval number R20160727).

### Genetic constructs

The CAR construct was modified from a lentiviral transfer vector pRRLSIN-WPRE-GFP (Addgene #12252) wherein WPRE was substituted with an EF-1α minimal promoter and GFP with a CAR. The second-generation CAR comprised of a single-chain variable fragment (scFv) with a G4S linker, a CD8 hinge, a CD28 transmembrane domain, a CD28 costimulatory domain, and a CD3ζ signaling tail. The HER2 CAR consisted of anti-HER2 scFv (Clone FRP5) and the EphA3 CAR consisted of an anti-EphA3 scFv fragment derived from the anti-EphA3 antibody ifabotuzumab (formally KB004, or IIIA4) (patent number: WO2021092560A1). The empty vector (EV) control construct contained a GFP protein in place of a CAR.

### Flow cytometry

EphA3 cell surface antigen expression on tumor cell lines was determined by staining with a recombinant anti-EphA3 antibody (produced by ATUM) with the same variable scaffold and complementarity-determining regions (CDRs) as ifabotuzumab. This biosimilar antibody was used at 3.75 µg/mL and a mouse anti-human IgG BV711 secondary antibody (Becton Dickinson Biosciences). To determine the transduction efficiency of the human T cells, CAR cell surface expression was measured by labeling T cells with αG4S linker (Clone E7O2V, Cell Signaling Technology). T cells were labeled using αCD45 (Clone HI30) (BioLegend), αCD3 (Clone SK7) (BD Biosciences), αCD4 (Clone OKT4) (BioLegend), and αCD8 (Clone SK1) (BD Biosciences) antibodies. Viability dyes included LIVE/DEAD Fixable Yellow, Fixable Blue (Invitrogen), and Zombie NIR fixable viability dye (BioLegend).

To determine the effect of stimulation on the CAR T cells, human CAR T cells were co-cultured with either media (unstimulated control), αCD3/CD28 Dynabeads (Life Technologies), or U251CL target cells at a 1:1 E:T ratio at 37°C, 5% CO^2^ for 18 hours. The cells were analyzed for markers of activation by labeling with αCD3, αCD4, αCD8, αCD137 (Clone BC96) (BioLegend).

Bleeds on mice were performed to phenotype the human T cells in circulation. 30–50 µl of blood was processed by red cell lysis buffer (Ammonium Chloride 156mM, Sodium bicarbonate 11.9mM and EDTA 0.097mM, WEHI), then labeled with αCD45, αCD3, αCD4, αCD8, αCD45RA (Clone HI100) (BD Biosciences) and αCD197 (Clone G043H7) (BioLegend) antibodies. All samples were prepared in MACS buffer (PBS+0.02% (v/v) FCS+2 mM EDTA).

Cells were analyzed using the FortessaX20 flow cytometer (Becton Dickinson) or Cytek Aurora Borealis (Cytek Bio) and FlowJo software (Becton Dickinson, V.10.9).

### Cell surface proteomics

Cell surface proteomics was performed on each sample in quadruplicate using an amino-oxy-biotin labeling technique, previously described.^31^ Briefly, fresh tumor samples or cell lines were mechanically dissociated into single-cell suspension and exposed surface sialic acid residue were biotinylated with sodium meta-periodate, analine, and amino-oxy-biotin (Biotium). Nuclei were removed by centrifugation and biotinylated proteins enriched with streptavidin-agarose (Pierce). Proteins were digested off the beads with Trypsin Gold and peptides were collected by centrifugation, and tryptic fractions were analyzed for mass spectrometry analysis with an Impact II operated in a data-dependent mode. Raw files were analyzed using MaxQuant. The database search was performed using the UniProt Homo Sapiens database and the relative abundance of proteins was determined using intensity based absolute quantitation (iBAQ) (total precursor intensities divided by the theoretically observable number of peptides).

### Immunohistochemistry analysis of patient tissues and quantification using QuPath

Formalin-fixed paraffin-embedded (FFPE) tissue blocks were sectioned, dewaxed, and subjected to heat-mediated antigen retrieval using a microwave in 0.01 M sodium citrate buffer (pH 6.0). After cooling, sections were washed and endogenous peroxidases were quenched using 1% hydrogen peroxide for 10 min. Nonspecific antibody binding was blocked using 10% normal goat serum from Vector Laboratories (Burlingame). Sections were incubated overnight at 4°C with rabbit anti-EphA3 (LSBio, Shirley, Massachusetts, USA) or control rabbit IgG, then with secondary antibody for 35 min at RT using goat anti-rabbit-biotin. Detection steps were performed using the Vectastain Elite ABC HRP kit (Vector Laboratories) according to the manufacturer’s recommendations, followed by a reaction with the DAB peroxidase substrate kit (Vector Laboratories). Sections were counterstained using Mayer’s hematoxylin (Chem-Supply) and mounted in DPX mountant (Sigma-Aldrich). Stained slides were scanned on a NanoZoomer HT (C9600-01) (Hamamatsu) at 40X and image files were imported to QuPath V.0.4.3 for analysis. Whole slide images were annotated for areas of interest (to exclude histologically normal tissue and areas of bland necrosis). Within these areas, pixels above a threshold of 0.24 were classified as EphA3 positive, and the area above the threshold calculated.

### T-cell transduction

Lentivirus was produced via transfection of HEK293T cells using the plasmids: pMD2G-VSVg (Addgene #12259), pMDLg/pRRE (Addgene #12251), pRSVREV (Addgene #12253), and CAR transfer vector pRRL-SIN-EF1α-CAR using FuGENE-6 (Promega) as per the manufacturer’s protocol. Human CD4^+^ and CD8^+^ T cells were selected from healthy donor PBMCs using CD4^+^ isolation and CD8^+^ positive selection kits (EasySep, StemCell Technologies). Selected T cells were activated with αCD3/CD28 Dynabeads (Life Technologies) for 48 hours. Two rounds of transduction of the human T cells were performed on consecutive days (48 and 72 hours post activation) on retronectin-coated plates (Takara Bio), which were spinoculated for 1 hour at 1000 G and 22°C.

### Cytotoxicity assay

Target cells were seeded in triplicate in 96 flat bottom plates before co-culture with human CAR T cells in the presence of 50 µM propidium iodide (PI, Calbiochem). PI uptake into target cells was measured as a surrogate for cell death, and images were captured every hour for 24 hours by the IncuCyte (Sartorius, Models S3, SX5). Results were analyzed using IncuCyte software (Sartorius, V.2021A).

### Cytometric bead array

Cytometric bead arrays were conducted to assess the cytokine production of the human CAR T cells in response to stimulation, according to manufacturers’ instructions, and as previously described.^32^ Human CAR T cells were assessed with no stimulation (cells alone), with αCD3/CD28 Dynabeads (Life Technologies), or with a human GBM cell line (U251CL). The levels of interferon gamma (IFN-γ) and tumour necrosis factor alpha (TNF-α) were measured using Cytokine Bead Array (CBA) Flex sets (Becton Dickinson) following the manufacturer’s protocol. Measurements were performed using the FACS Verse (BD Biosciences), and analysis was carried out using FCAP array software V.3.0 (BD Biosciences).

### Mice

Orthotopic xenograft models were performed on NOD.Cg-*Prkdc^scid^IL2*rgtmWjl/SzJ (JAX NSG strain code 614) (Jax Flora) (NSG) female mice that were bred under specific pathogen-free conditions at WEHI Kew Facility. Mice were 7–9 weeks of age at primary tumor implantation and 17–19 weeks of age at tumor rechallenge. Mice were monitored at the animal facility at WEHI (Parkville, Victoria, Australia) under the approved WEHI AEC #2022.063. Mice were euthanized at the ethical endpoint.

### Xenograft models

To establish tumors, NSG mice were intracranially injected with 50,000 tumor cells (U251CL or SU-DIPG36GL) in 3 µL at 2 mm right of the bregma at the coronal suture to a depth of 3 mm from the cranium into the brain using a stereotactic frame. Mice were given subcutaneous injections of (10 mg/kg) enrofloxacin (Headway Animal Health) on the day of procedure followed by 3 days postsurgery. 1 week postsurgery, mice were imaged via bioluminescence imaging (described below) to estimate tumor burden, then allocated randomly into treatment groups. Mice were then treated with a single dose of 5–10^6^ CAR T cells at a 1:1 ratio of CD4^+^:CD8^+^ cells via intravenous injection through the tail vein. Mice were euthanized at the ethical endpoint as defined by the approved ethical guidelines (WEHI AEC #2022.063).

### Bioluminescence imaging

Bioluminescence imaging was performed weekly to monitor tumor growth over time. Mice were injected intraperitoneally with 3 mg VivoGlo Luciferin (Promega) in 200 μL, then anesthetized with an inhalant isoflurane dose. Imaging was performed on the IVIS Lumina III Series Hardware (Perkin-Elmer) and analysis was performed on the Living Image Software (Perkin-Elmer, V.4.7.2). Average radiance values photons per second per centimeter squared per steradian, abbreviated as p/s/cm^2^/sr was used as a measurement of tumor burden.

## Results

### Cell surface proteomics identifies EphA3 as a promising target for adult and pediatric brain cancers

**Figure 1:**
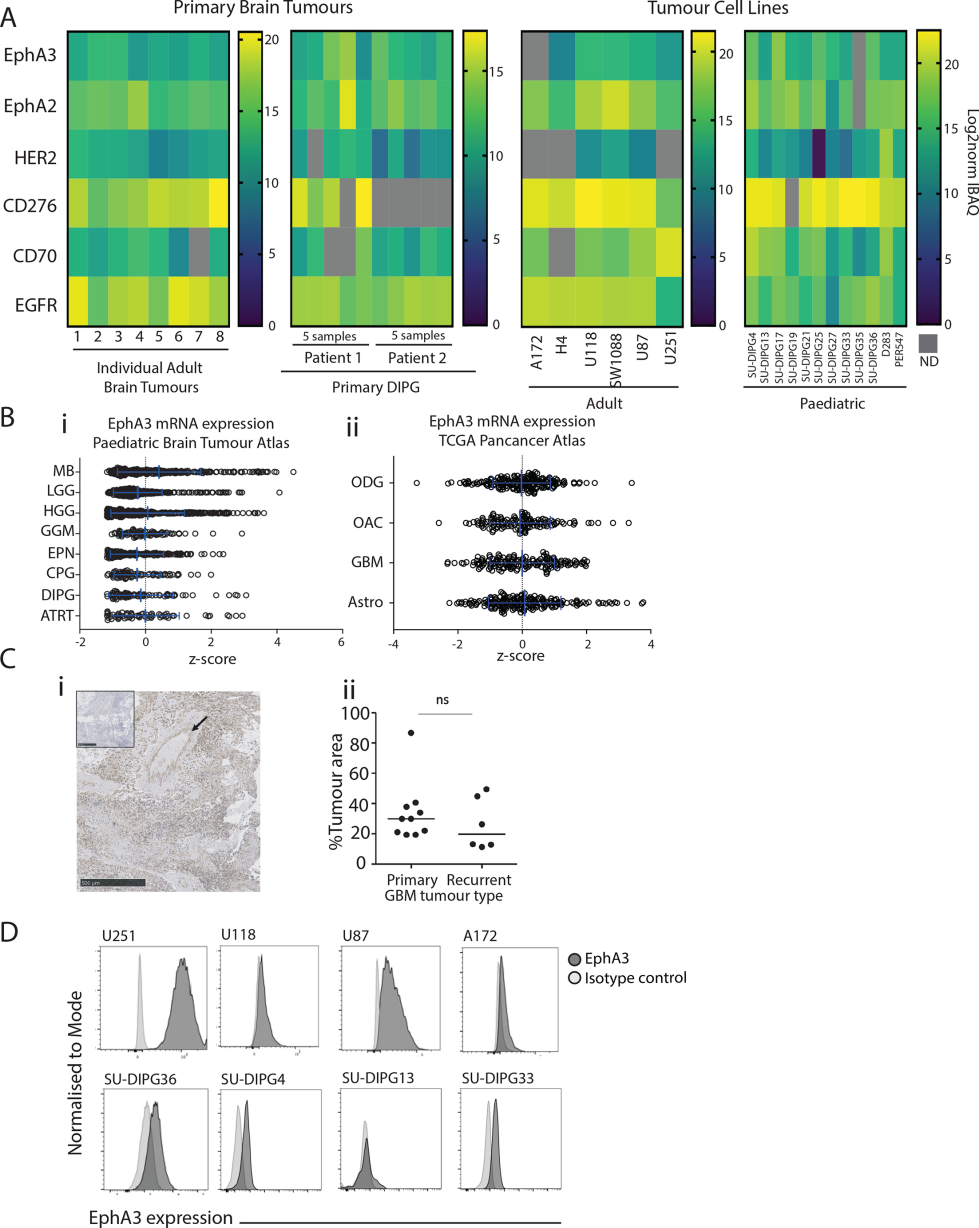
EphA3 is overexpressed in adult GBM and pediatric DIPG. (**A**) Heatmap of cell surface expressed human brain tumor targets EphA3, EphA2, HER2, CD276, CD70, and EGFR, as detected using mass spectrometry and ranked by iBAQ log transformed detection intensities. Heatmaps from right to left depicts primary brain tumors (n=8), rapid autopsy DIPG tumors (n=2, with five samples tested from each), adult brain tumor cell lines (n=6), and pediatric brain tumor cell lines (n=12). (**B**) EphA3 messenger RNA expression (z-score) in pediatric brain tumors (Primary Brain Tumor Atlas) and adult brain tumors (TCGA Pancancer Atlas). (**C**) Immunohistochemistry analysis of EphA3 expression in patient GBM tumor tissues. Image shows a representative EphA3-stained tissue (main) with isotype control (inset). Arrow highlights perivascular staining. Summary graph shows automated quantification of positively stained area across all analyzed tissues (n=10 primary tumors, n=6 recurrent tumors, bars represent median). Difference between primary and recurrent disease was not significant by Mann-Whitney test. (**D**) Flow cytometry analysis of EphA3-BV711 labeling of adult brain tumor (top row) and pediatric. Astro, astrocytoma; ATRT, atypical teratoid/rhabdoid tumor; CPG, craniopharyngioma; DIPG (bottom row) cell lines; DIPG, diffuse intrinsic pontine glioma; EphA3, Ephrin type-A receptor 3; EPN, ependymoma; GBM, glioblastoma; GGM, ganglioma; HGG, high-grade glioma; iBAC, intensity based absolute quantification; LGG, low-grade glioma; MB, medulloblastoma; mRNA, messenger RNA; OAC, oligoastrocytoma; ODG, oligodendroglioma; TCGA, The Cancer Genome Atlas.

Cell surface proteomics is an effective approach in the identification of cell surface immunotherapy biomarkers.^31^ For novel target discovery, it needs to be applied to fresh primary brain tumors. Through clinical collaboration, we were able to access either fresh primary brain tumors in adults or rapid autopsy DMG brain tumors in pediatrics. These samples were then comprehensively analyzed for their cell surface proteome (figure 1A). Analysis of the cell surfaceome in pediatric and adult tumors revealed EphA3 as a prominent cell surface protein across various brain tumor subtypes (online supplemental table 1), especially in the context of comparison to existing immunotherapeutic targets in clinical development EphA2, HER2, CD276, CD70, and EGFR (figure 1A). We next sought to perform similar comparisons in a range of preclinical models including adult high-grade glioma cell lines (A172, H4, U118, SW1088, U87, U251), DIPG cell lines (SU-DIPG4, −13 to –17, −19 to –21, −25 to –27, −33 to –35, and −36), and medulloblastoma cell lines (D283, PER547) (figure 1A). Similar to primary samples we determined EphA3 to be broadly and robustly expressed across all CNS tumor models examined, including high and low grade brain tumors. From this analysis, we were able to determine that the abundance of EphA3 was comparable to other notable CAR T cell targets currently undergoing clinical examination, including; CD276 [NCT04385173], EphA2,^33^ HER2,^34^ CD70 (NCT05353530), and EGFR (NCT03638167).

Having detected cell surface expression of EphA3 on primary tumors and cell lines in pediatric DMG and medulloblastoma, we next sought to determine if targeting EphA3 was applicable to other pediatric CNS tumors. Examination of EphA3 mRNA expression in the pediatric CNS dataset from the Primary Brain Tumor Atlas (PBTA) clearly demonstrates groups of patients with EphA3 expression at comparable levels to DIPG in other pediatric brain cancer types, including Atypical Teratoid/Rhabdoid Tumor (ATRT), craniopharyngioma, ependymoma, ganglioma, high and low grade gliomas as well as medulloblastoma (figure 1B(i)).^35^ Similar examination of an adult mRNA dataset from The Cancer Genome Atlas Pancancer Atlas clearly demonstrates groups of patients with EphA3 expression at comparable levels to glioblastoma in other brain cancer types including oligodendroglioma, oligoastrocytoma, and astrocytoma (figure 1B(ii)). Collectively these data suggest that there is a subpopulation of patients across CNS tumors whereby targeting EphA3 with a CAR may have clinical benefit.

We next examined EphA3 expression on tumor biopsy tissues collected in the setting of primary or recurrent disease to determine the heterogeneity of expression within the tumors (figure 1C). EphA3 was expressed by all tumors examined, with staining of tumor parenchyma and perivascular areas observable (figure 1C(i)). Automated quantification using QuPath revealed that the total area of viable tumor tissue stained positive for EphA3 ranged from 11% to 87%, with no significant difference between primary or recurrent tumors (figure 1C(ii)).

We next sought to examine EphA3 cell surface antigen expression on brain tumor models by staining with a recombinant anti-EphA3 antibody with the same variable scaffold and CDRs of ifabotuzumab (WO2008/112192A2). This biosimilar antibody was used to determine the cell surface expression of EphA3 across multiple GBM lines (U251CL, U118, U87, and A172) and the DIPG cell lines (SU-DIPG36GL, SU-DIPG4, SU-DIPG13, SU-DIPG33) (figure 1D). Notably, expression was highest in the U251CL and U87 lines, while relatively lower levels were observed in U118, A172, SU-DIPG36GL, SU-DIPG4, SU-DIPG13, SU-DIPG33, underscoring the potential for EphA3-targeted CAR therapies in diverse glioma contexts. Recognizing that for an effective therapy to be developed in brain cancer, it will also likely need to target the glioma stem cells (GSC) population. We, therefore, examined the co-expression of common GSC markers (CD133, CD44, CD70, L1CAM, S100A4) using our cell surface proteomics dataset across the adult and pediatric primary tumors as well as U251CL and SU-DIPG36GL cell lines (online supplemental figure 1). This analysis demonstrated that both the U251CL and SU-DIPG36GL cell lines share similar levels of GSC markers present in the adult and pediatric primary tumors, reflecting their suitability for the preclinical examination of EphA3 CAR targeted therapies.

### Human EphA3-CAR T cells exhibit robust anti-glioma function

**Figure 2:**
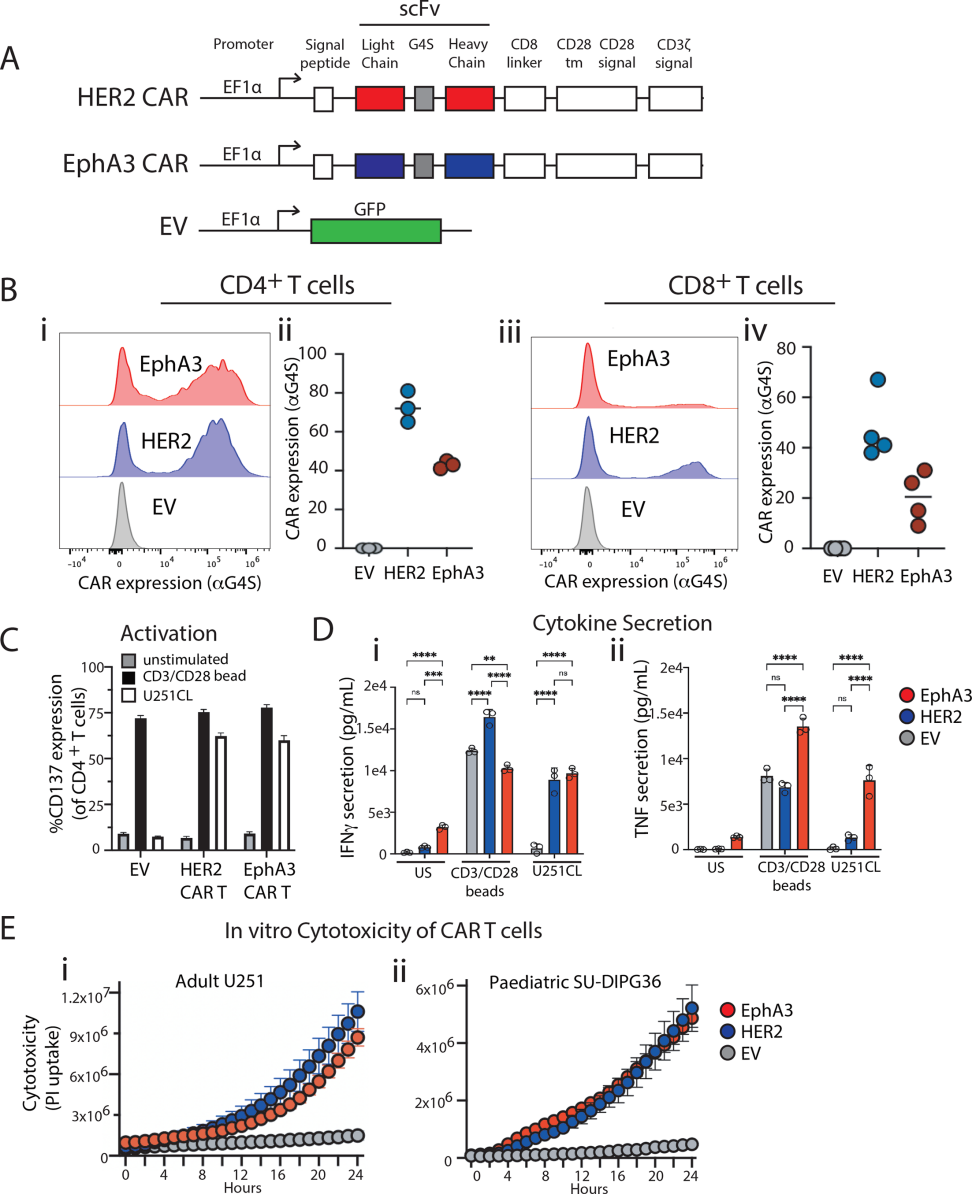
EphA3 CAR T cells are functional against adult and pediatric glioma. (**A**) Schematic of second generation CAR constructs; (**B**) flow cytometry of cell surface expression of EphA3 (red) and HER2 (blue) CAR, as detected using anti-G4S protein tag antibody, and the percentage of CAR+ of T-cell population shown for three independent human donors; activated CD4^+^ EphA3-CAR T cells after 16 hour co-culture with U251CL tumor cells, or CD3/CD28 beads as positive control showing (**C**) %CD137 expression as detected using flow cytometry and (**D**) IFNγ and TNF secretion (pg/mL) into the culture. Transduction efficiencies of CD4+ GFP+ (empty vector) or CD4+ G4S+ CAR T cells shown in (**C**) and (**D**) for each group are as follows: EV 62%, HER2 94%, EphA3 79%. Shown is mean of triplicates (n=3± STDEV, *p<0.05 using two-way analysis of variance); (**E**) IncuCyte killing assay of 2.5:1 ratio of CD8^+^ CAR T cells co-cultured with adult U251CL glioma cells or pediatric SU-DIPG36GL tumor cells over 24 hours. Transduction efficiencies for CD8+ GFP+ (empty vector) or CD8+ G4S+ CAR T cells for each group are as follows: EV 41%, HER2 43%, EphA3 25%. CAR, chimeric antigen receptor; EphA3, Ephrin type-A receptor 3; HER2, Human epidermal receptor protein 2; IFNγ, interferon gamma; scFv, single-chain variable fragment; TNF, Tumour necrosis factor.

Given the high expression of EphA3 on both glioma tumor cells and the perivasculature surrounding the tumor, we proceeded to create and test a second-generation EphA3-targeted CAR. Reconstructing the ifabotuzumab antibody into a scFv, we then integrated it into a standard second-generation CAR backbone consisting of a CD8α hinge, a CD28 transmembrane, and co-stimulation domain, followed by a CD3ζ signaling domain (figure 2A). A G4S linker served as dual purpose to not only link the heavy and light variable chains but also act as a protein tag that could be used to detect cell surface CAR expression. To examine the efficacy of the EphA3 CAR, we first purified and activated CD4^+^ and CD8^+^ T cells separately from healthy donors before lentiviral transduction of the EphA3 CAR or the HER2 CAR as a positive control and clinical benchmark.^10^ An EV construct that contained GFP was used as a negative control. On day 6 post-transduction, we used flow cytometry detection of G4S to measure EphA3 and HER2 CAR cell surface expression in both CD4^+^ and CD8^+^ T cells (figure 2B). While EphA3 CAR expression was observed at lower levels compared with HER2 CAR expression, particularly in the CD8^+^ T cells (figure 2B(iv)), cell surface CAR expression was detected and relatively consistent across multiple independent human donors (figure 2B).

Having validated EphA3 CAR expression in primary T cells, we next examined its activity in vitro in co-culture assays. As expected CD4^+^ EphA3 CAR T cells co-cultured with GBM U251CL target cells for 18 hours exhibited antigen-specific activation as measured by the cell surface upregulation of CD137 similar to the HER2 CAR (figure 2C). Further characterization of the EphA3 CAR T cells in co-cultures with U251CL targets demonstrate antigen-specific secretion of IFNγ, which was comparable to HER2 CAR controls (figure 2D(i)), and interestingly we observed significantly increased TNF secretion by EphA3 CAR T cells (figure 2D(ii)). We were keen to evaluate the antigen-specific cytotoxic function of the EphA3 targeted CAR T cells. To do this we performed a 24-hour cytotoxicity IncuCyte assay against both the adult GBM cell line (U251CL) and pediatric DIPG cell line (SU-DIPG36GL) (figure 2E). CD8^+^ EphA3 CAR T cells exhibited cytotoxicity against U251CL and SU-DIPG36GL cells in an antigen-specific manner, equivalent to the positive control HER2-CAR T cell benchmark, with no observable cytotoxic effect observed in the EV negative controls. Collectively, these data clearly demonstrate that the EphA3 CAR expressed in primary human CD4^+^ and CD8^+^ T cells, whereby it can function in an antigen-dependent manner to signal to induce activation, cytokine secretion, and exert cytotoxic effect on target brain tumor cell lines.

### Human EphA3 CAR T cells are effective against pediatric DMG in xenograft mouse model

**Figure 3:**
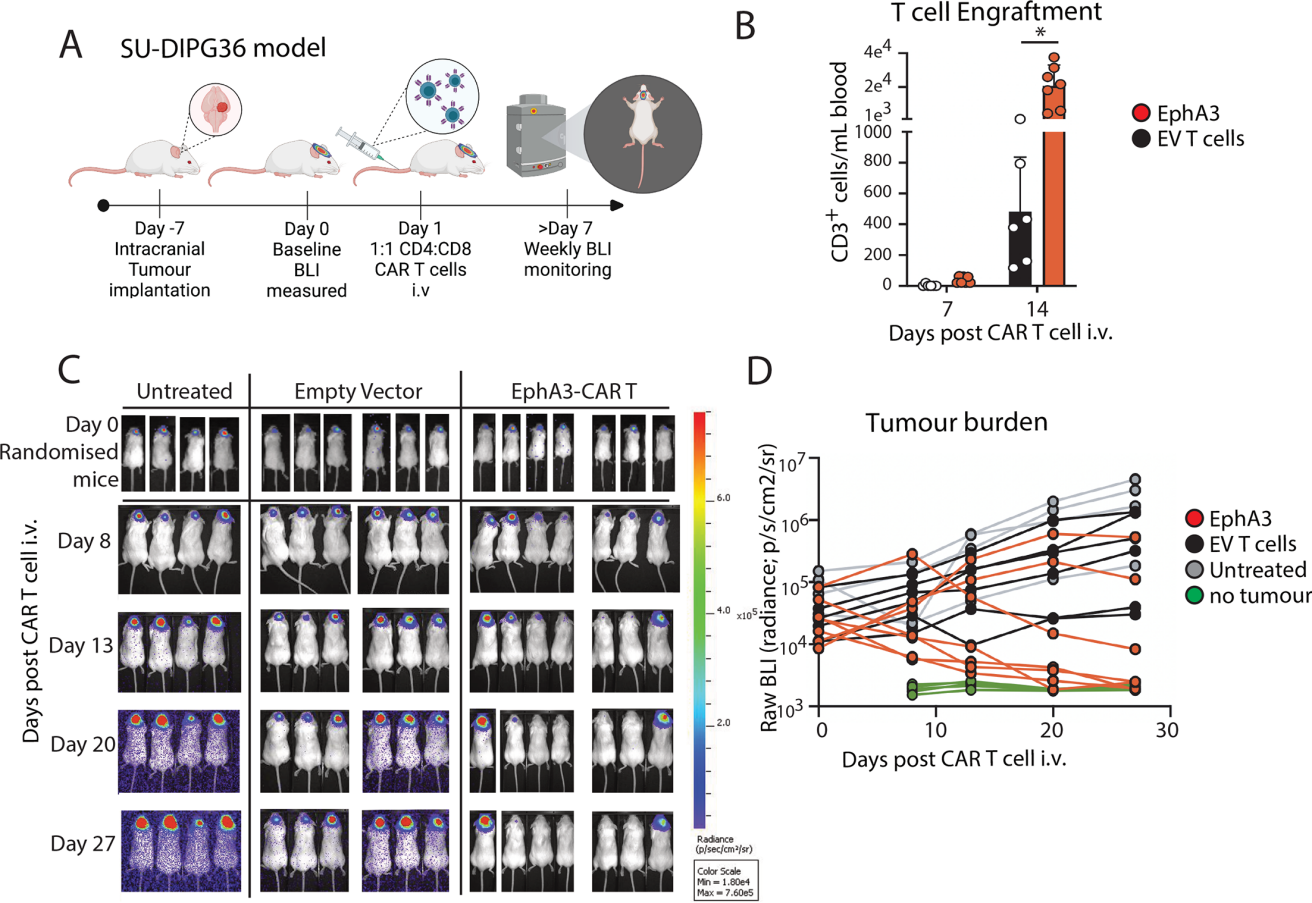
EphA3 CAR T cells are effective against intracranial pediatric DMG tumors. (**A**) Schematic representation of intracranial in vivo experiments using pediatric SU-DIPG36GL glioma model. (**B**) Quantitation of human CD3^+^ T cells detected in peripheral blood of NSG mice bearing SU-DIPG36GL intracranial tumors treated with empty vector (EV) (n=6) or EphA3-targeted CAR T cells (n=7), shown day 7 and 14 post CAR T cell intravenous (i.v) injection. Shown is mean of biological replicates±STDEV. T cells are the same as used in figure 2E. Transduction efficiencies of CD4+ GFP+ (empty vector) or CD4+ G4S+ CAR T cells used for treatment of each group are as follows: EV 52%, HER2 75%, EphA3 71%. (**C**) Bioluminescence images of NSG mice depicting tumor engraftment at day 0 of CAR T-cell treatment, and subsequent tumor growth up to day 21 post treatment. (**D**) Tumor progression relative to baseline at day 0 post CAR T cell i.v injection of mice shown in (**C**). Each line represents a single mouse, and a cohort of mice who never received tumors imaged as a negative control to show the baseline BLI and experiment was ended at day 27 post CAR T cell injection. Students t-test at experimental endpoint day 27 between individual groups and p values are as follows: untreated versus EphA3 p<0.05 (p=0.009), and no tumor versus EphA3 not significant (p=0.3), EV versus EphA3 (p=0.06). BLI, bioluminescence imaging; CAR, chimeric antigen receptor; DMG, diffuse midline gliomas; EphA3, Ephrin type-A receptor 3.

Having established that the EphA3 CAR was functional in vitro, we next sought to assess the therapeutic efficacy of EphA3-targeted CAR T cells in an in vivo model of intracranial pediatric DMG using NSG mice (figure 3A). To do this SU-DIPG36GL cells were intracranially injected into the right cerebral cortex, as previously described.^36^ Following tumor implantation growth was assessed using bioluminescence imaging (BLI) 7 days postoperative, with quantification of tumor burden used to assign mice into treatment cohorts using a negative hypothesis bias. The following day, mice received a single intravenous dose of 1×10^7^ transduced T cells total at a 1:1 ratio of CD4^+^ and CD8^+^ T cells for either negative control EV T cells or EphA3 CAR T cells. Mice were then monitored weekly using BLI to track tumor growth. Peripheral blood was taken on days 7 and 14 post T cell IV infusion. At day 7 post transfer, low levels of circulating CD3^+^ T cells were observed in the circulating blood of both treatment groups, indicating engraftment had occurred (figure 3B). However, by day 14, there was a significant increase in CD3^+^ T cells in EphA3 CAR-treated mice, indicative of an antigen-specific response (figure 3B). As expected, all untreated mice and mice treated with EV T cells demonstrated consistent tumor growth until the ethically defined endpoint (figure 3C,D). Complete tumor clearance was observed in 5 out of 7 (71.4%) mice treated with EphA3 CAR T cells by day 20 post treatment (figure 3B) and maintained to day 27 after which all mice were euthanized.

### Human EphA3 CAR T cells are effective against adult GBM in a mouse xenograft model

**Figure 4:**
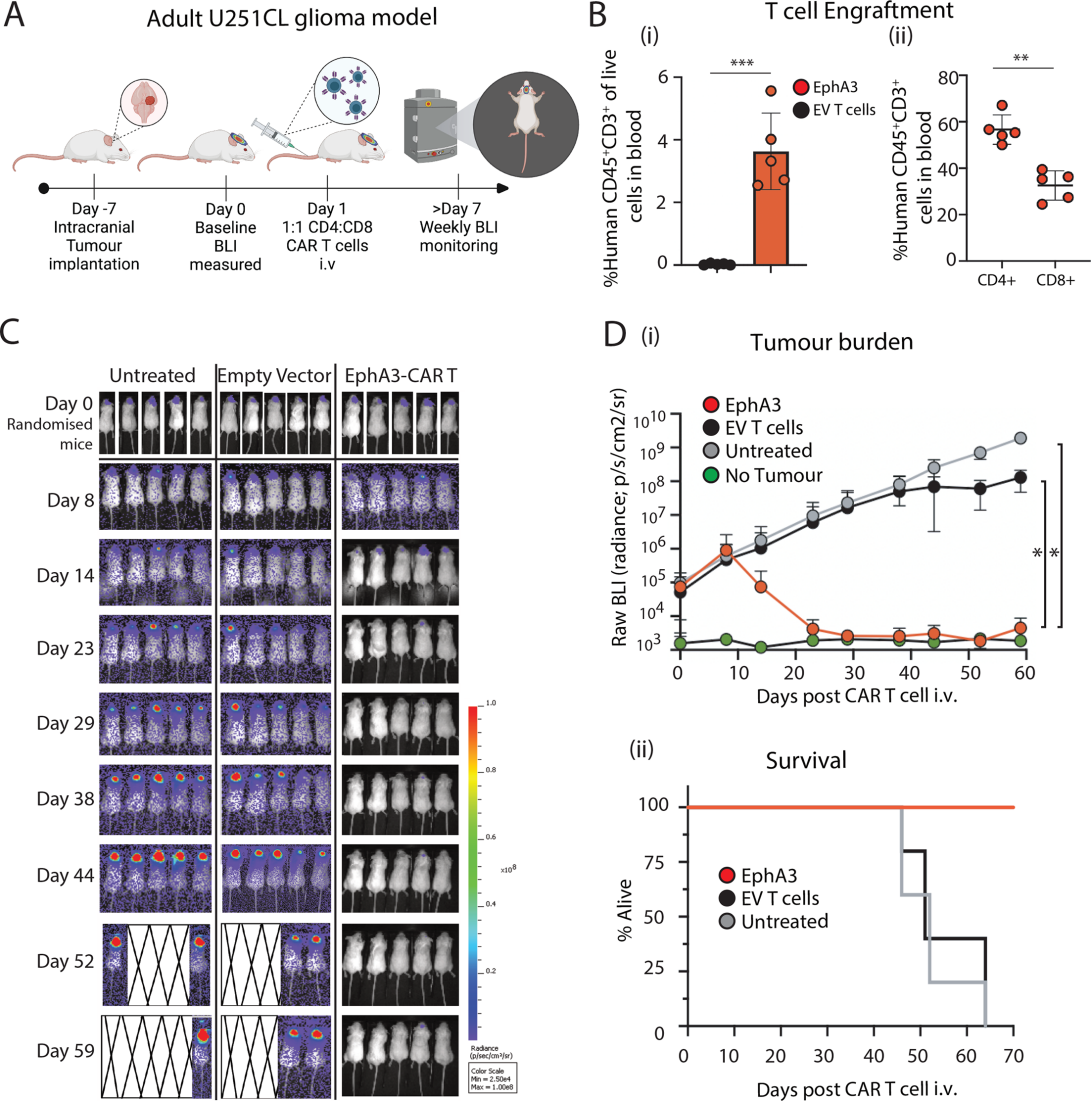
EphA3 CAR T cells are curative against orthotopic implanted glioblastoma in NSG mice. (**A**) Schematic representation of intracranial orthotopic in vivo experiments using adult U251CL glioma model. (**B**) Quantitation of human CD45+ CD3+ T cells detected in peripheral blood of NSG mice bearing U251CL intracranial tumors, including (i) percentage of CD45+ CD3+ cells in blood in EV (n=5) or EphA3-targeted CAR T cell treated mice (n=5), and (ii) the percentage of CD4 and CD8 EphA3-CAR T cells as a proportion of CD45+ CD3+ cells detected in EphA3-CAR T cell treated mice shown at day 16 after CAR T cell intravenous (i.v) injection. Transduction efficiencies of CD4+ GFP+ (empty vector) or CD4+ G4S+ CAR T cells used for treatment of each group are as follows: EV 62%, HER2 91%, EphA3 79%. Transduction efficiencies for CD8+ GFP+ (empty vector) or CD8+ G4S+ CAR T cells for each group are as follows: EV 42%, HER2 35%, EphA3 3%. (**C**) Bioluminescence images of NSG mice depicting tumor engraftment at day 0 of CAR T cell treatment and subsequent tumor growth up to day 59 post treatment. On day 52 post CAR T cell treatment, one mouse in untreated group was immediately culled following imaging due to reaching ethical endpoint. (**D**) (i) Average BLI radiance demonstrating tumor burden (p/s/cm2/sr). Shown is the mean±STDEV of EphA3 (n=5), EV treated (n=5) mice and tumor bearing, but untreated (n=5) compared with mice not implanted with tumors (no tumor, n=4). (ii) Kaplan-Meier survival curve out to day 70. Data is representative of two independent human biological donors. Statistical comparison of individual groups was performed using Students *t*-test. *p<0.05, **p<0.005, ***p<0.0005. BLI, bioluminescence imaging; CAR, chimeric antigen receptor; EphA3, Ephrin type-A receptor 3.

Given the promising results in the pediatric DMG model, we next investigated the in vivo efficacy of EphA3-targeted CAR T cells against an adult GBM orthotopic model (figure 4A). Similar to the DIPG model, U251CL cells were injected intracranially, tumors were measured for size with BLI 7 days later, assigned to treatment groups, and treated intravenously with a single dose of 1×10^7^ transduced EV or EphA3 CAR T cells at a 1:1 ratio of CD4^+^ and CD8^+^. Examination of peripheral blood at day 16 showed significant engraftment of human CD45^+^CD3^+^ EphA3 CAR T cells compared with EV (figure 4B(i)). Interestingly, despite the delivery of CD4^+^ and CD8^+^ EphA3 CAR T cells at a 1:1 ratio, we observed a consistently significant increase in CD45^+^CD3^+^CD4^+^ compared to CD45^+^CD3^+^CD8^+^ cells in the EphA3 CAR T cell mice (figure 4B(ii)).

As anticipated, mice that remained untreated or treated with nontargeting EV T cells demonstrated consistent tumor growth until they reached the ethical endpoint at 9 weeks post-tumor implantation (figure 4C–D). Mice treated with EphA3 CAR T cells exhibited tumor eradication within 4 weeks after treatment, leading to a prolonged complete response and significantly increased overall survival (figure 4C–D).

### CAR T cells form a memory population and can protect mice from a second antigen challenge

**Figure 5:**
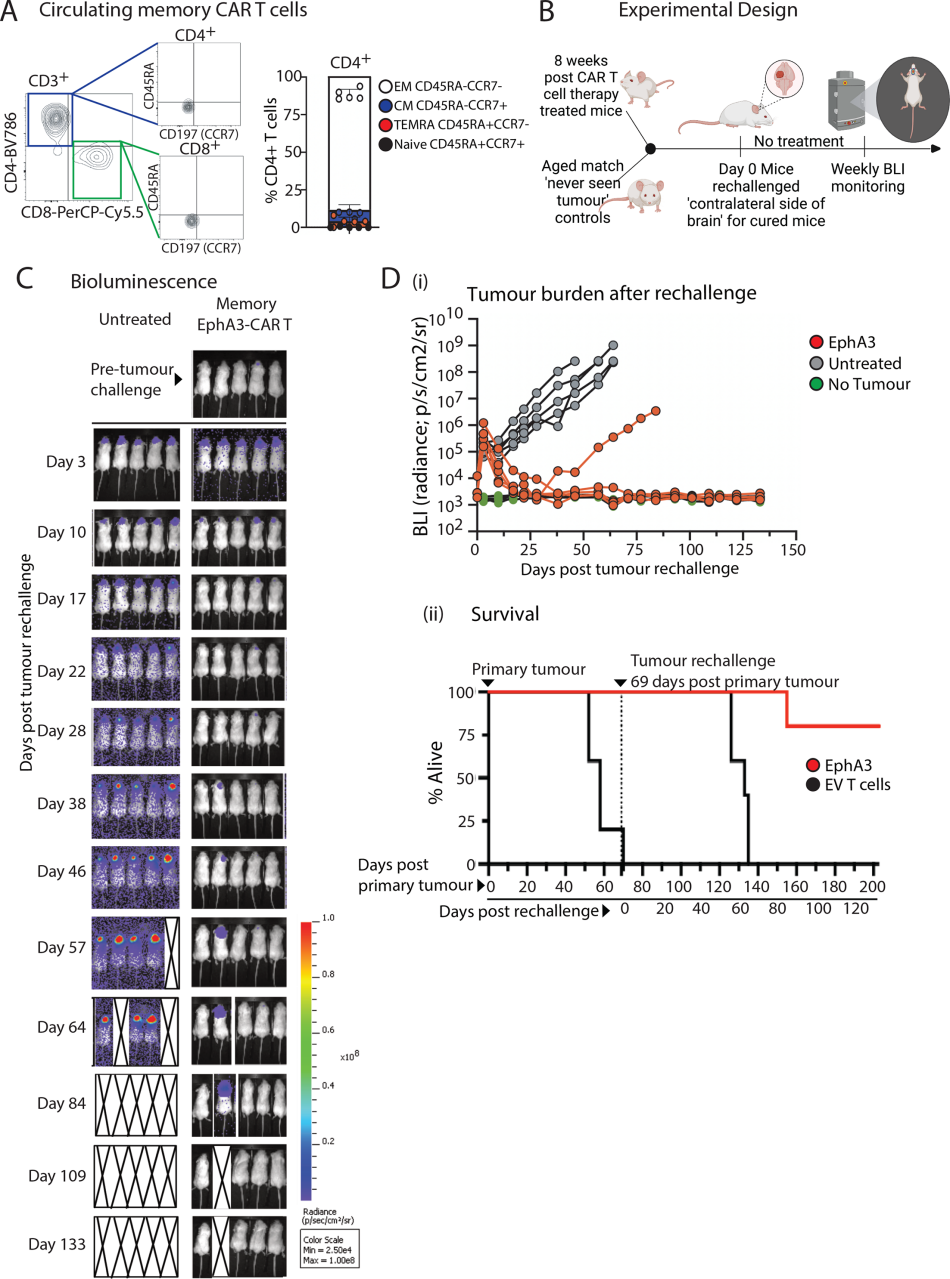
Memory EphA3-CAR T cells provide long-term protection from rechallenge of orthotopic glioblastoma tumors. (**A**) EphA3 CAR T cell treated NSG mice (from figure 4) demonstrated circulating CD4+ and CD8+ T cells as analyzed by flow cytometry. Representative flow cytometry plots are shown of blood cells labeled with CD45RA-FITC and CC47-BV510, backgated on live CD45+ cells. (**B**) Schematic representation of rechallenge intracranial experiment using EphA3-CAR T cell treated mice shown in (**A**) and age-matched treatment-naïve controls. (**C**) Bioluminescence images of NSG mice depicting tumor engraftment at day 3 post tumor rechallenge implantation and subsequent tumor growth. On day 57 post-tumor rechallenge, one mouse in untreated group was immediately culled following imaging due to reaching ethical endpoint. (**D**) Quantitation of tumor size and Kaplan-Meier survival curve of the mice shown in (**C**). BLI, bioluminescence imaging; CAR,chimeric antigen receptor; EphA3, Ephrin type-A receptor 3.

Given the potent and complete response by EphA3 targeted CAR T cells in the U251CL mouse model, we monitored the mice for the presence of circulating human T cells at 9 weeks post CAR infusion (figure 5A). Human CD3^+^ T cells were observed in all mice, with a predominantly effector memory phenotype as determined by the lack of both CD45RA^+^ and CCR7^+^ cell surface markers (figure 5A). Therefore, we speculated that circulating or resident memory CAR T cells could continue to confer protective function on a secondary challenge.

After confirming that the mice had remained tumor-free for 5 weeks, we assessed the functional recall capabilities of the remaining human EphA3 CAR T cells by conducting a contralateral orthotopic rechallenge experiment. Using healthy aged-matched treatment naive control mice (figure 5B), we performed intracranial injections of U251CL cells into the contralateral side of the brain from the initial injection site. No further CAR T cell treatments were given, with BLI tumor growth was monitored weekly.

Within 3 days following the tumor rechallenge, we observed BLI tumor measurements in all mice, confirming successful implantation (figure 5C). The treatment-naive mice showed continuous tumor growth, reaching the ethical endpoint between 7 and 9 weeks post-implantation (figure 5D). Strikingly, complete tumor clearance occurred within 2-3 weeks of implantation in all five mice (100%), mice previously treated with EphA3 CAR T cells. Of these, one mouse exhibited a relapse 38 days post-implantation, despite demonstrating full BLI clearance of tumor and eventually reached the ethical endpoint at 86 days post-tumor implantation. The remaining four mice were observed to be tumor free for over 200 days post-tumor implantation.

## Discussion

In our study, the utilization of cell surface proteomics to validate EphA3 as a target for glioma treatment has showcased its effectiveness in detecting promising immunotherapy biomarkers and targets directly from fresh primary brain tumors. Our analysis, which included freshly resected high-grade gliomas and rapid autopsy pediatric tumors (figure 1A), revealed a significant presence of EphA3, a result consistent with prior findings.^22 37^ EphA3 demonstrated comparable expression levels when compared with established CAR T cell targets such as CD276 (NCT04385173), EphA2,^33^ and HER2,^34^ underscoring its potential for clinical translation. Previous studies have also shown high expression of EphA3 in glioblastoma and very low expression in normal brain,^22^ and a lack of expression on vital organs including heart, liver, kidney, and lung^23^ strongly suggesting that off-tumor on-target effects are unlikely.^17^

In addition, toxicity studies demonstrated the absence of cellular expression of EphA3 in normal human and cynomolgus monkey tissues (Hagey *et al* 2011, Blood ASH Annual meeting) and the first study to identify EphA3 as a target also showed no detection in normal brain.^38^ In a related publication using the same EphA3-targeted clone as a monoclonal antibody in advanced hematological malignancies demonstrated mild and transient toxicities.^24^

Further, the strongest evidence for the safety of this EphA3 clone (ifabotuzumab) targeting EphA3 is provided by an existing clinical trial, which assessed a radiolabelled antibody against EphA3.^25^ In this study, Gan and colleagues have demonstrated the biodistribution and pharmacokinetics of ^89^Zr-labeled ifabotuzumab and showed a favorable safety profile and lack of uptake in healthy brain.^25^ The absence of normal tissue uptake of the tracer indicated ideal characteristics for a range of therapeutic approaches. Concordant with our study (figure 1), previous studies have also shown EphA3 is prominently expressed around the tumor vasculature of GBM xenografts, a known stem cell niche in GBM^39^ and tumor stroma.^20^ Interestingly, consistent targeting of the tumor microenvironment was also highlighted in interim reports of the clinical trial using ifabotuzumab.^40^ However, it is important to consider that the kinetics of an antibody-drug-conjugate may differ from the cell therapy format, therefore an EphA3 CAR T cell safety trial should be conducted.

Moreover, extending our investigation to pediatric CNS tumors, the expression data from the Primary Brain Tumor Atlas confirmed that EphA3 is not only prevalent in high-grade gliomas but also in a spectrum of other pediatric brain tumors suggesting the broader applicability of EphA3 targeted therapies across CNS malignancies (figure 1B). This widespread expression warrants further exploration into EphA3 as a universal therapeutic target beyond high-grade gliomas, potentially benefiting a wider pediatric patient cohort. While the expression of CD276, EGFR, and EphA2 are higher than EphA3 (figure 1A), the expression levels of EphA3, and the localization within the perivasculature warranted further development.

We also validated the cell surface presence of EphA3 using the anti-human EphA3 antibody ifabotuzumab, confirming its expression on various glioma cell lines through flow cytometry (figure 1C). Notably, the highest expression levels were detected in U251CL and U87 glioma lines, while it was lower in others such as SU-DIPG36GL suggesting a possible correlation between EphA3 expression levels and the efficacy of targeted CAR T cell therapies. Such heterogeneity in expression, as further quantified in primary GBM tumors (figure 1D), highlights the need for personalized approaches in CAR T cell therapy, potentially guided by EphA3 expression levels to maximize therapeutic outcomes. Additionally, given the comparable expression levels observed in other DIPG cell lines as shown in figure 1, EphA3 is likely to be an effective target across a broader range of DIPG variants, not just the SU-DIPG36GL cells.

The robust anti-glioma effect displayed by EphA3-CAR T cells, particularly in our in vivo models, aligns with the therapeutic efficacy seen in other studies targeting surface antigens in solid tumors, including lung cancer and melanoma.^18^ Our findings, which show substantial tumor eradication in both pediatric and adult glioma models, validating the potential of EphA3 as a CAR therapeutic target (figures3  4). In addition, EphA3 is expressed on distinct stromal/fibroblast-like cell types in the tumor microenvironment (TME) that promote growth and angiogenesis and genetic knockdown of EphA3 reduces angiogenic capacity.^18^ The complete responses observed in a significant proportion of our models underscore the promise of this approach, although acknowledging the challenges posed by immune evasion and the tumor microenvironment that may necessitate combination therapies or iterative CAR designs. In addition, the CAR T cell responses to adult glioma (with high EphA3 expression) were more effective than against a glioma target expressing lower antigen levels, highlighting the importance of target antigen density in patient selection in clinical trial design.^41^

The discovery of new targets for CAR T cells remains crucial for the development of more effective and safe treatments, particularly as antigen loss and resistance to therapy emerge. The field has recognized that monotherapies alone may be insufficient for long-term regression, even in liquid tumors. For instance, CD22-targeted CARs have been developed to overcome resistance to CD19 CAR T cell therapy, offering an alternative strategy with lower toxicity.^42^

Our preclinical data demonstrate that an EphA3-targeted CAR T cell is effective against EphA3-expressing high-grade-gliomas. Given EphA3’s status as a tumor-associated antigen, it serves as an ideal target for use in combination with other CAR T cell therapies, either as a multitargeted approach or within a logic-gated system. Future studies are anticipated to adopt personalized and bespoke strategies tailored to individual tumors, aiming to enhance the coverage of DIPG or DMG antigens for more efficient targeting Additionally, EphA3-targeted CAR T cells could be used in combination with other immunomodulatory drugs, such as an anti-CD47 antibody that could address the immunosuppressive tumor microenvronment.^43^

Furthermore, the formation of a memory T-cell population following EphA3 CAR T cell treatment suggests the potential for long-term immunity against glioma recurrence, a critical aspect often lacking in conventional treatments (figure 5). The rechallenge experiments provide compelling evidence of the recall function of EphA3 CAR T cells, with a notable percentage of mice exhibiting tumor clearance on secondary tumor implantation without further CAR T cell treatment. Our study demonstrates that EphA3-targeted CAR T cells, after clearing primary tumors, persist for extended periods (over 8 weeks) in NSG mice. These CAR T cells are capable of being reactivated on re-exposure to tumor cells, which is a hallmark of memory T cell functionality. This persistence and recall ability of CAR T cells is well documented in the literature as indicative of memory T cell responses.^44 45^ The observed long-term persistence and reactivity of these CAR T cells align with the established definitions and functional characteristics of memory T cells.^46^

Furthermore, recent studies have emphasized that CAR T cells can develop memory-like properties, contributing to sustained anti-tumor immunity.^47^ This has been observed in both preclinical models and clinical settings, where CAR T cells exhibit the ability to persist and maintain functionality over extended periods, akin to endogenous memory T cells.

In conclusion, while the data strongly support the clinical exploration of EphA3 CAR T cells in glioma treatment, it is paramount to consider the intertumor and intratumor heterogeneity that may influence therapeutic outcomes.

Ongoing and future studies should aim to refine CAR T cell therapies based on tumor-specific and patient-specific characteristics to enhance efficacy and reduce potential resistance or relapse. Further clinical trials, informed by our preclinical data, are essential to determine the real-world applicability and long-term benefits of EphA3-targeted CAR T cell therapies in glioma patients.

## supplementary material

10.1136/jitc-2024-009486 online supplemental file 1

## Data Availability

Data are available upon reasonable request.
