# Supplementary material for: EphA3-targeted chimeric antigen receptor T cells are effective in glioma and generate curative memory T cell responses
Source: J Immunother Cancer. 2024 Aug 6;12(8):e009486. doi: 10.1136/jitc-2024-009486 (PMC11308882; doi:10.1136/jitc-2024-009486)

<u>**Supplementary Figures**</u>

Lertsumitkul et al manuscript titled: **EphA3-targeted chimeric antigen receptor T cells are curative in adult and paediatric models of glioma and generate protective memory T cell responses.**

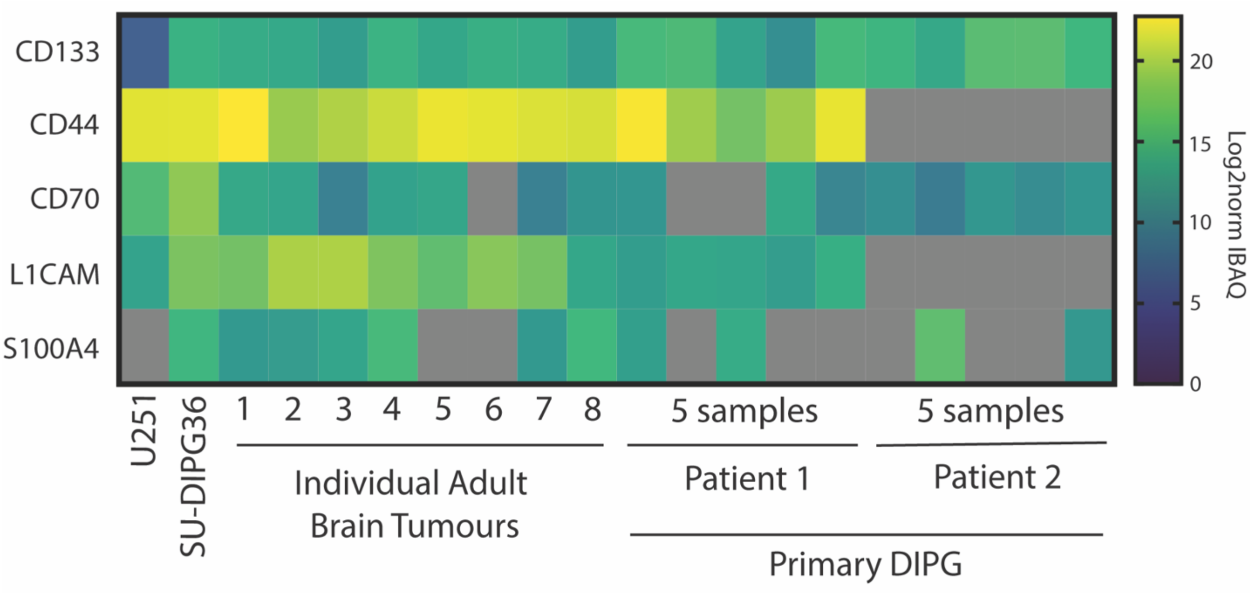

Supplementary Figure 1:
Heatmap of cell surface proteins CD133, CD44, CD70, L1CAM, S100A4, as detected using mass spectrometry and ranked by IBAQ log transformed detection intensities. Heatmaps from right to left depicts U251 and SU-DIPG36 cell lines, followed by primary brain tumours (n=8), rapid autopsy DIPG tumours (n=2, with 5 samples tested from each).

Supplementary Table 1:

Pathology	Identifier	Type	Surgery	Grade	Age	Sex	Ki 67	IDH Status	ATRX	p53	1p/19 co-deleted	TERT	EGFR
Gemistocytic astrocytoma	1	Primary	Debulking	II	50	F		mutated					
Oligodendroglioma	2	Primary	Debulking	II	24	M	<4 %	mutated	unmutated	mutated	yes		
Oligodendroglioma	3	Primary	Debulking	III	46	M		mutated			yes	mutated	not amplified
GBM	4	Primary	Biopsy	IV	58	M	<5 %	wild type	unmutated	mutated			
GBM	5	Primary	Debulking	IV	43	F		wild type				mutated	not amplified
GBM	6	Primary	Debulking	IV	77	F	20 %	wild type	unmutated	mutated			
GBM	7	Primary	Debulking	IV	50	M	10 %	wild type				mutated	not amplified
Gliosarcoma	8	Primary	Debulking	IV	45	M	<5 %	wild type	unmutated	mutated			
GBM	U251CL	Cell Line		IV	75	M		wild type		mutated		mutated	

Supplementary Figure 2 – Flow Cytometry Back-gating strategies

Gating strategy for Figure 2B (CAR expression)

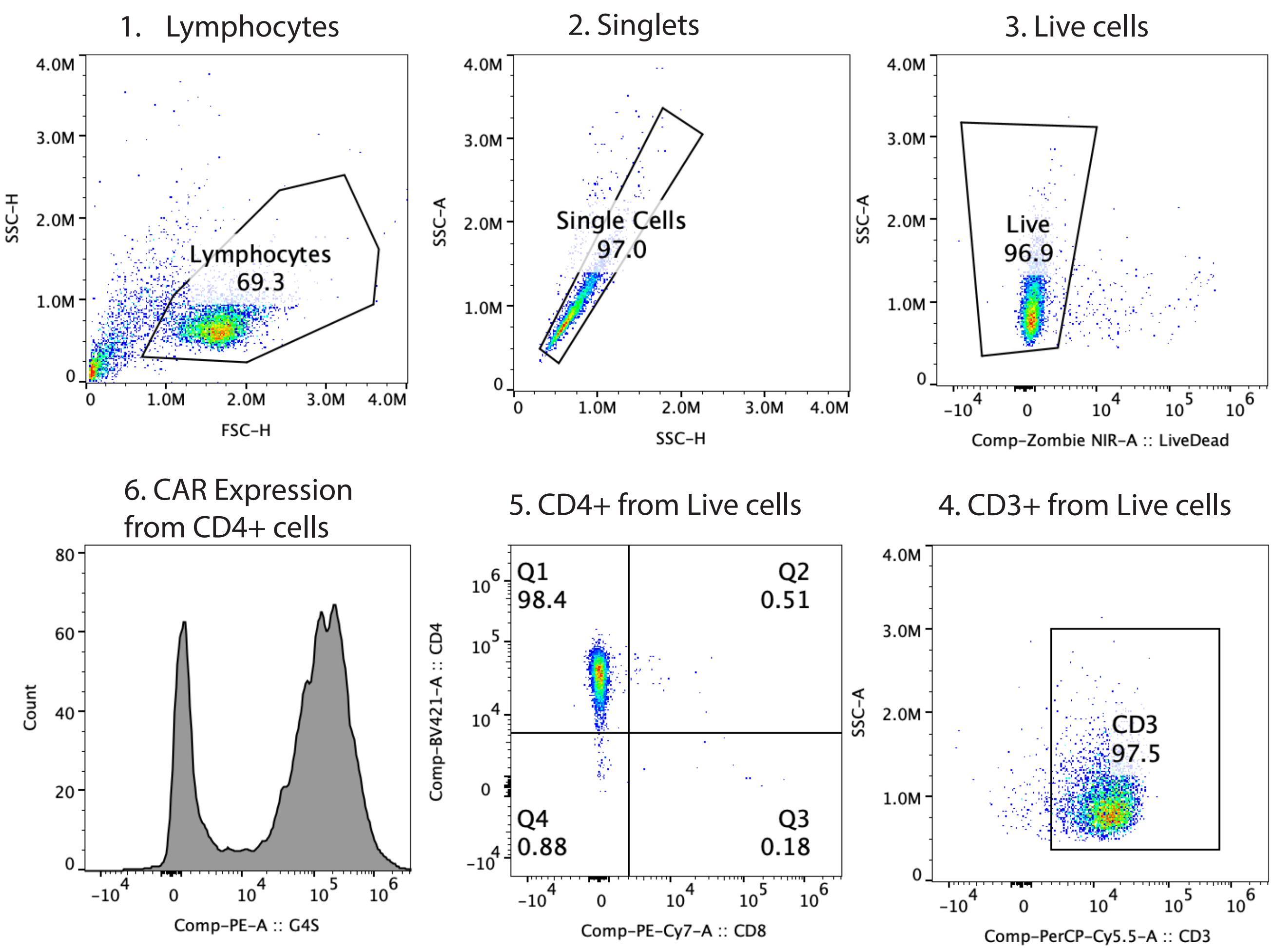

Gating strategy for Figure 2C (CD137 expression)

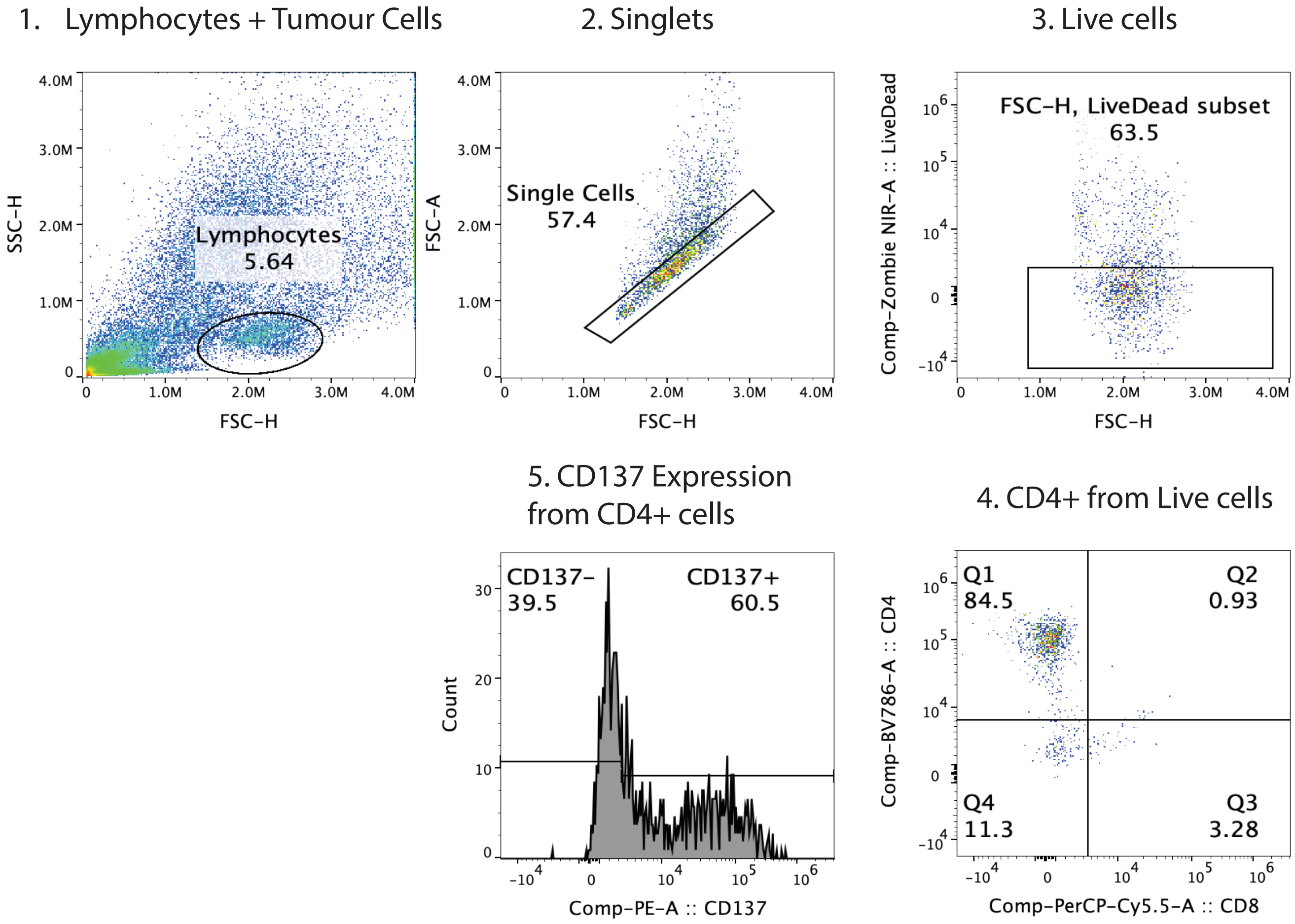

Gating strategy for Figure 3B (T Cell Engraftment)

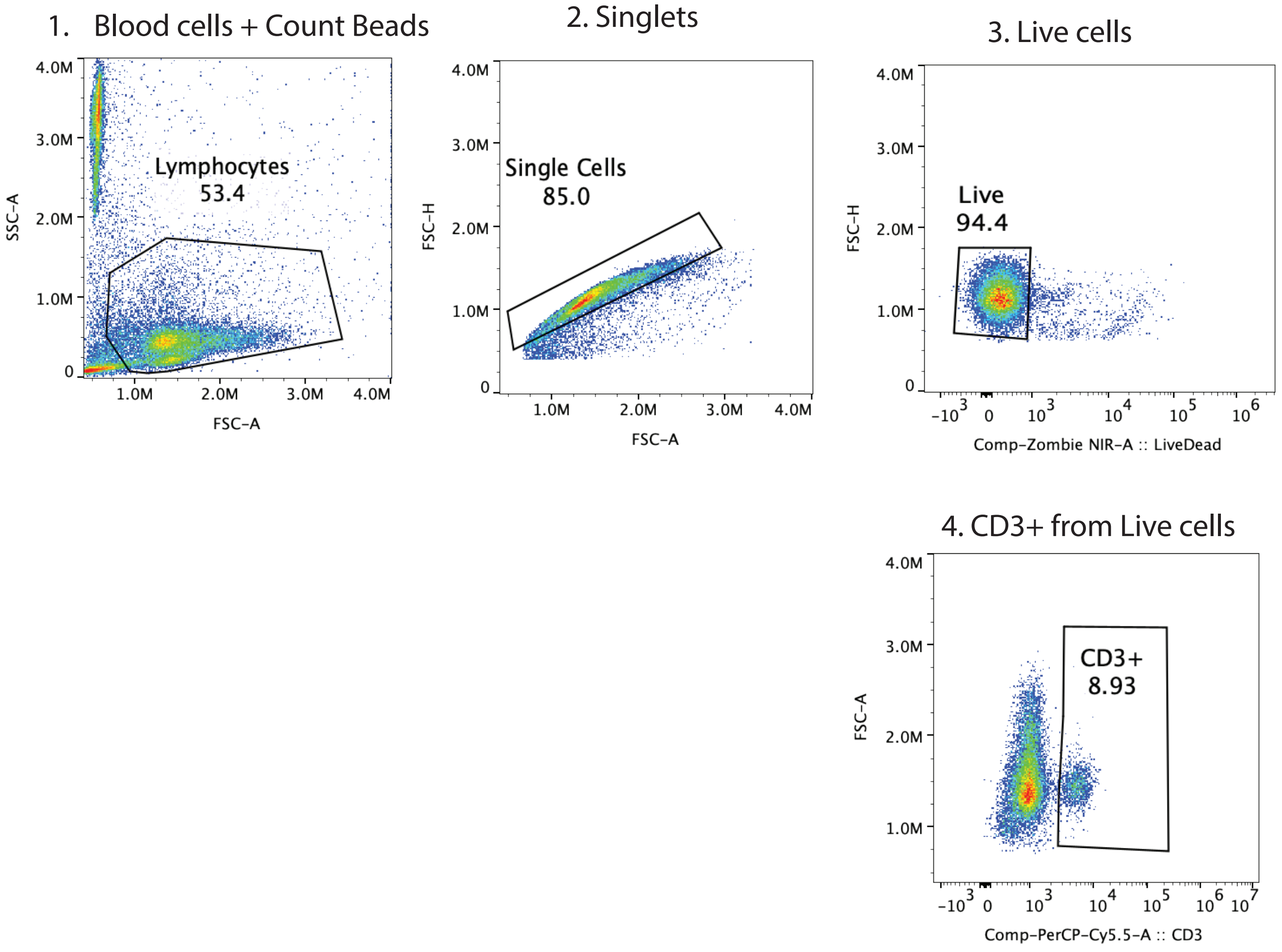

Gating strategy for Figure 4B (T Cell Engraftment)

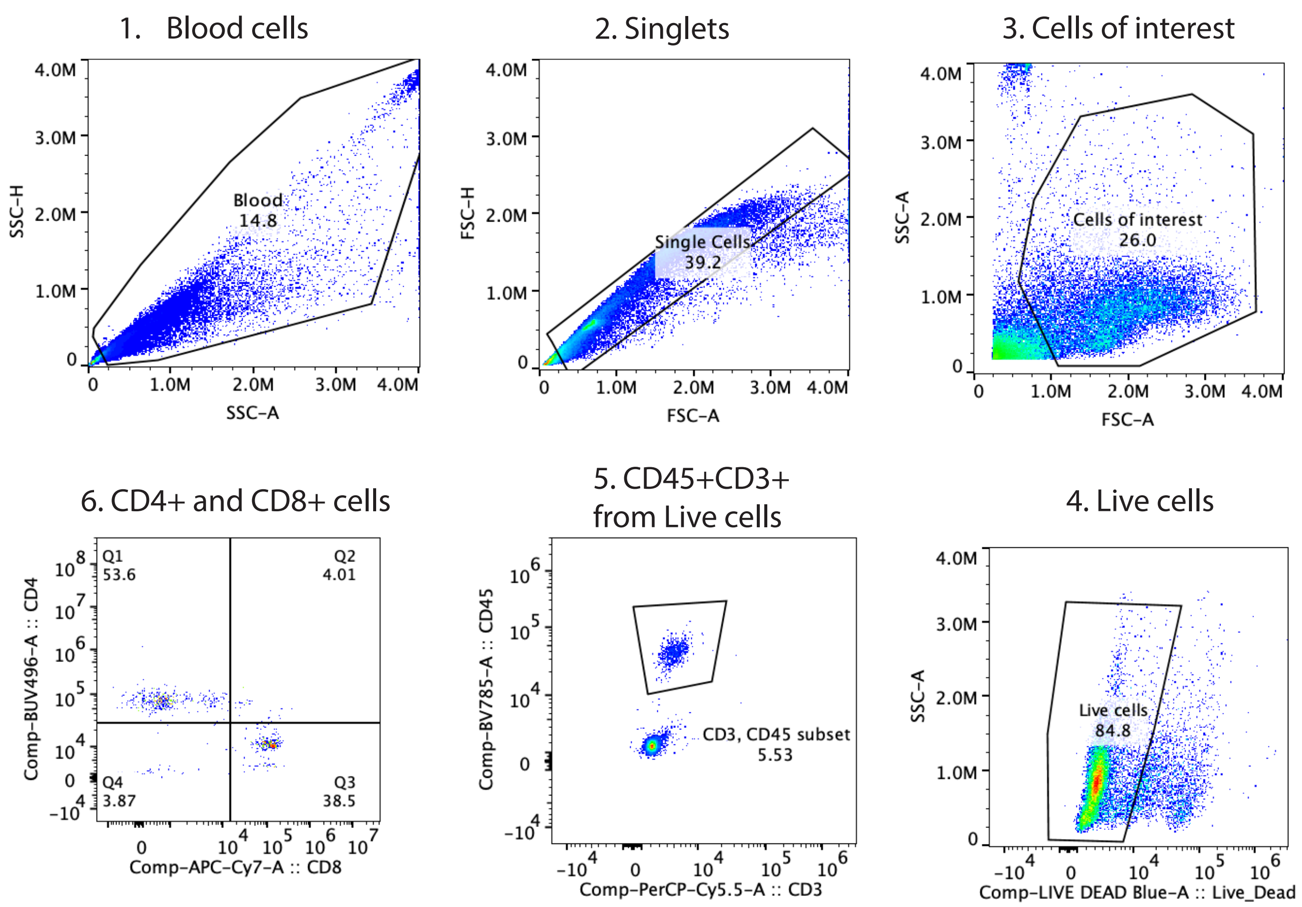

Gating strategy for Figure 5A
(Pre-Tumour Rechallenge Blood Circulating memory CAR T cells)

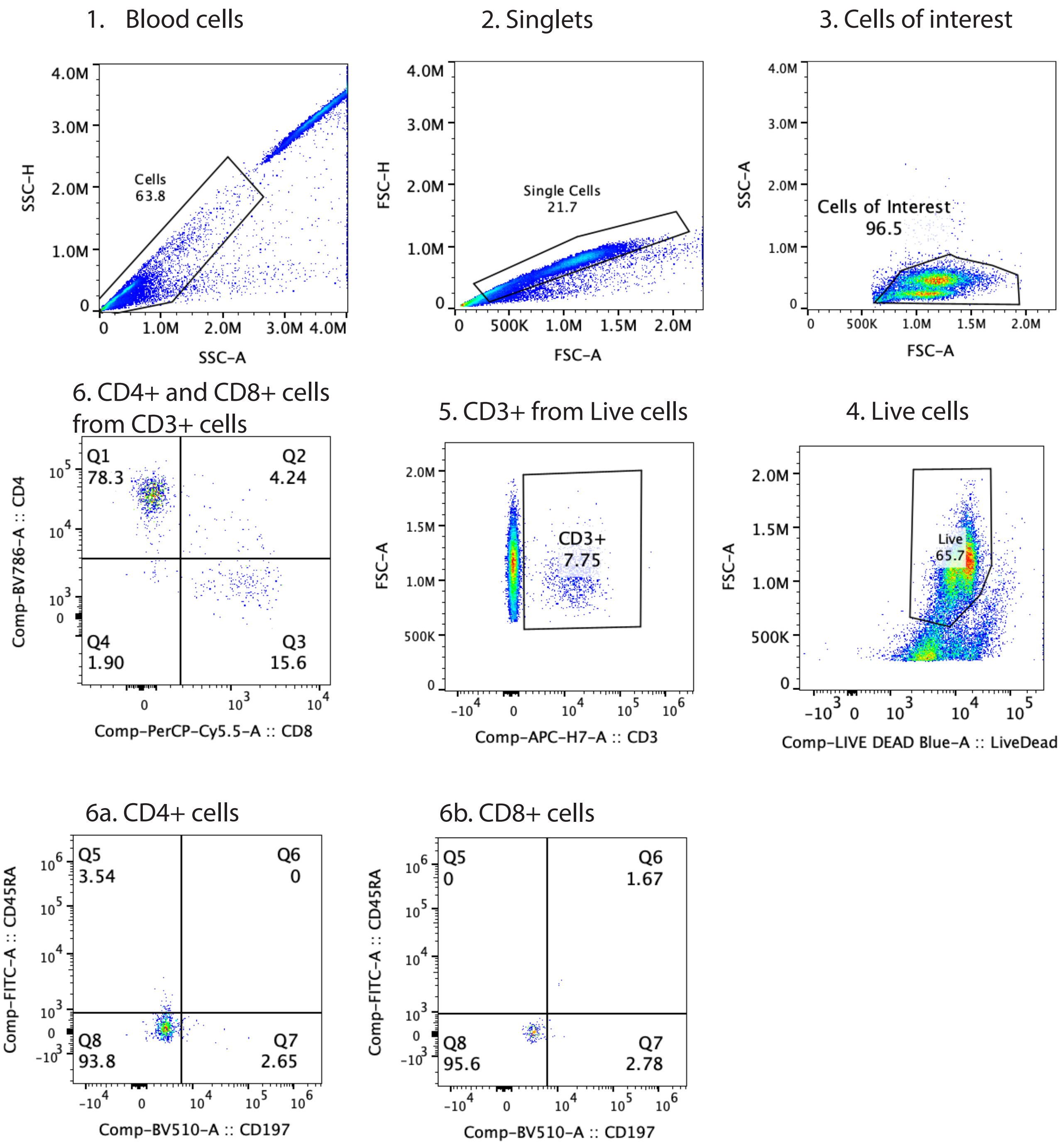

Supplement: online supplemental file 1 [file jitc-12-8-s001.pdf]
